# Molecular Mechanism of Antidepressant Effects of Active Ingredients in Traditional Chinese Medicine: The Role of Autophagy

**DOI:** 10.31083/AP45685

**Published:** 2025-08-11

**Authors:** Li-juan Zhang, Dan Chen, Kai-yong Xu, Rui-rui Shang, Xiao-yu Liu, Zi-fa Li, Kang-feng Wang, Min Zhang

**Affiliations:** ^1^No. 3 Department of Encephalopathy, Affiliated Hospital of Shandong University of Traditional Chinese Medicine, 250011 Jinan, Shandong, China; ^2^Experimental Center, Shandong University of Traditional Chinese Medicine, 250355 Jinan, Shandong, China; ^3^College of Rehabilitation Medicine, Shandong University of Traditional Chinese Medicine, 250355 Jinan, Shandong, China; ^4^Department of Vertigo, Shandong Provincial Hospital Affiliated to Shandong First Medical University, 250021 Jinan, Shandong, China

**Keywords:** depression, antidepressant, active ingredients of traditional Chinese medicine, autophagy, molecular mechanism

## Abstract

In clinical practice, selective serotonin reuptake inhibitors (SSRIs), a kind of Western medicine, are the primary treatment for depression, a complex mental illness. However, these treatments are associated with significant adverse reactions. With their many benefits and distinctive features, such as all-encompassing intervention and general control through several targets, processes, and pathways, the active components in traditional Chinese medicine (TCM) hold great promise for the treatment of depression. Autophagy plays a crucial role in the pathophysiology of depression, and its regulation has emerged as a potentially crucial management tactic. However, comprehensive evaluations of the link between depression and mitochondrial autophagy, as well as the therapeutic potential of TCM’s active components, remain limited. This review examined recent literature on autophagy and its role in depression, along with studies on the effects of TCM active ingredients. Furthermore, it highlighted the limitations of current research to offer insights to guide future fundamental studies and clinical treatments for depression.

## Main Points

1. Major depressive disorder (MDD) is a prevalent and debilitating mental illness characterized by persistent sadness, loss of pleasure, suicidal tendencies, and various physical symptoms.

2. Autophagy plays an important role in the pathological development of MDD.

3. The active ingredients of traditional Chinese medicine can treat MDD by regulating autophagy.

## 1. Introduction

Major depressive disorder (MDD), commonly referred to as depression, is a prevalent and debilitating mental illness characterized by persistent sadness, loss of pleasure, suicidal tendencies, and various physical symptoms. Due to its high lifetime incidence, recurrent nature, severe impairment, and chronic progression, depression is currently ranked as the fourth leading cause of disease burden worldwide by the World Health Organization [1]. The most used pharmacological treatments in clinical practice are selective serotonin reuptake inhibitors (SSRIs) and others. SSRIs exert their antidepressant effects by blocking the reuptake of 5-hydroxytryptamine (5-HT) at presynaptic membranes, specifically inhibiting 5-HT transporters, thereby extending and intensifying the effects of 5-HT [2, 3]. However, these medications are linked to significant side effects, including headaches, nausea, weight gain, and persistent sexual dysfunction. Moreover, most therapies have high non-response rates and delayed effects [4, 5, 6]. As a result, the development of safer and more effective antidepressants has become an urgent priority.

Due to its multi-component, multi-target, and multi-pathway effects, traditional Chinese medicine (TCM) represents a viable alternative for antidepressant treatment. Active compounds found in TCM, such as oridonin, baicalin, and quercetin, have shown strong antidepressant benefits with minimal adverse side effects [7], highlighting the vast potential of TCM in antidepressant treatment. For instance, research has found that Xiaoyao Pill, a Chinese patent medicine, can alleviate depressive symptoms through a mechanism of action involving regulation of DNA methylation [8].

Autophagy, an evolutionarily conserved metabolic process involving protein and organelle degradation, is crucial for maintaining cellular homeostasis [9]. Dysregulated autophagy plays an important role in the pathogenesis and development of MDD, making autophagy modulation a novel therapeutic strategy for its treatment. However, there is currently no systematic summary on the relationship between autophagy and MDD, nor of the antidepressant effects of TCM active ingredients through autophagy regulation.

This review summarized the mechanism of autophagy, its relationship with MDD, and the mechanism of active ingredients of TCM in treating depression via autophagy regulation, providing a scientific basis for future basic research and clinical applications.

## 2. Overview of Autophagy

Autophagy is the primary degradation pathway in eukaryotic cells, responsible for breaking down aging organelles and large molecular substances, such as invading viruses and bacteria, thereby removing obstacles to cell growth [10, 11]. Autophagy includes three main types: macroautophagy, microautophagy, and molecular chaperone-mediated autophagy. These processes rely on lysosomes to degrade and recycle cytoplasmic components.

Microautophagy can be further classified into selective, non-selective, and endosomal autophagy. Its key mechanism involves the lysosomal membrane forming arm-like or petal-like protrusions that envelop cytoplasmic material or organelles for degradation [12]. Heat shock cognate protein 70 (HSC70) and other proteins selectively break down proteins with *KFERQ*-like domains, facilitating molecular chaperone-mediated autophagy. These proteins are transferred to lysosomes via lysosomal receptors for degradation [13]. Cytoplasmic components primarily reach lysosomes by mega-autophagy, also referred to simply as autophagy. This process involves the engulfment and degradation of organelles and cellular components inside a double-membrane framework. This complex, multi-step membrane transport process can be divided into the stages of initiation, nucleation, elongation, fusion, and degradation [14]. Mitophagy is a specific form of autophagy dedicated to tagging, removing, and recycling damaged or depolarized mitochondria. In addition, mitophagy plays a role in clearing excess organelles, such as sperm mitochondria, following fertilization [15].

## 3. Changes in Autophagy in MDD

### 3.1 Clinical Studies

In patients with MDD, dysregulated expression of autophagy-related genes has been identified in blood monocytes [16], along with abnormalities in the *AKT1* and mammalian target of rapamycin (mTOR) signaling pathways, both of which are essential for autophagy regulation [17, 18]. Over and beyond that, alterations in extracellular vesicle mRNA in postpartum depression patients have been found to be enriched in genes potentially associated with autophagy. Disrupted extracellular vesicle mRNA communication related to autophagy may contribute to the pathological development of postpartum depression [19]. A bioinformatics analysis further identified autophagy-related genes, such asG protein-coupled receptor 18 (*GPR18*), pyruvate dehydrogenase kinase 4 (*PDK4*), neuregulin 1 (*NRG1*), and EPH receptor B2 (*EPHB2*), as potential diagnostic markers for MDD, with *GPR18* possibly playing a role in its pathogenesis [20].

### 3.2 Preclinical Studies

Neuroinflammation is an essential part of the pathophysiology of depression. Evidence from animal models supports the involvement of autophagy dysregulation in the development of depression. The autophagy process is related to the activation of NOD-like receptor pyrin domain-containing 3 (NLRP3) inflammasomes. Mice with dysfunctional lysosomes in the autophagy-lysosome pathway may exhibit depressive-like behavior, increased synthesis of pro-inflammatory molecules, and interference with the breakdown of *NLRP3* inflammasomes [21]. Depression is exacerbated by the NLRP3 inflammasome, a multiprotein complex that triggers inflammatory cell death and caspase-1-mediated pro-inflammatory cytokines including interleukin-1β (IL-1β) [22].

Neuronal survival and function are also critical in depression. In a chronic restraint stress (CRS) depression model, autophagic cell death of neural stem cells (NSCs) led to hippocampal neuron damage in mice [23]. Similarly, in a corticosterone (CORT)-induced mouse model, excessive neuronal autophagy in the dentate gyrus (DG) increased the expression of autophagy related gene 5 (*ATG5*), resulting in excessive degradation of brain-derived neurotrophic factor (BDNF); It also significantly reduced the proliferation of NSCs, neural progenitor cell (NPCs), adult cells, as well as the migration and survival of recently formed neurons in DG. Knocking down ATG5 in neurons alleviated these effects and improved depressive-like behaviors in mice [24]. Notably, obesity has been demonstrated to reduce autophagy in a high-fat diet-induced obese mice model by boosting mTOR phosphorylation and inhibiting AMP-activated protein kinase (AMPK) phosphorylation, which results in depressive-like behaviors [25].

In summary, both clinical studies and animal models consistently reveal pathological autophagy imbalances in depression, indicating a close relationship between autophagy and depression. Regulating autophagy is one of the important key strategies for antidepressant treatment.

## 4. Molecular Mechanism of Active Ingredients in TCM

### 4.1 Flavonoids

Flavonoids, a class of naturally occurring polyphenolic compounds, are widely distributed throughout the plant kingdom [26]. One well-known flavonoid, quercetin, possesses immunoprotective, antiviral, antioxidant, neuroprotective, and cardioprotective properties [27]. A study demonstrated that quercetin inhibits NLRP3 inflammasome activation mediated by mitochondrial reactive oxygen species (mtROS) in microglia by promoting mitochondrial autophagy, thereby preventing neuronal damage and offering potential therapeutic effects for depression [28]. Another flavonoid, baicalin, which is extracted from the root of *Scutellaria baicalensis*, has antidepressant properties by enhancing NIX-mediated mitochondrial autophagy through direct AMPK binding and activation of the AMPK/peroxisome proliferator-activated receptor gamma coactivator 1-alpha (PGC-1α) pathway [29, 30].

Apigenin, a flavone found in plants such as chamomile, onions, fruits, and *salvia plebeia* [31], has shown antidepressant effects [32]. Zhang *et al*. [33] found that apigenin promoted autophagy via the *AMPK*/*mTOR* pathway in a CRS mouse model, alleviating depression. Silibinin, a key component of the silymarin complex extracted from the seeds of milk thistle (*Silybum marianum*) [34], upregulates BDNF/TrkB pathway activity and restores autophagy balance in the hippocampus, exerting antidepressant effects [35]. Kaempferol-3-O-sophoroside, a primary component of *Crocus sativus* (saffron), interacts with AMPK to stimulate BDNF production and autophagy, achieving antidepressant effects [36].

### 4.2 Terpenoids

Terpenoids, also known as isoprenoids, are natural isoprene-based compounds that play essential roles in metabolic processes across all organisms [37]. By preventing the connection between NLRP3 and NIMA related kinase 7 (NEK7), oridonin, a naturally occurring terpenoid present in several TCMs, reduces autophagy damage and neuroinflammation [38, 39]. It also activates autophagy to inhibit NLRP3 inflammasomes, thereby alleviating lipopolysaccharide (LPS)-induced depression [40].

Patchouli alcohol, a tricyclic sesquiterpene extracted from *Pogostemon cablin*, has anti-inflammatory effects [41]. Recent research indicates that it inhibits autophagy, repairs synapses, and restores autophagic flux in the hippocampus by activating the mTOR signaling pathway in chronic unexpected mild stress (CUMS) rats, hence demonstrating an antidepressant effect [42]. Andrographide, the main component of *Andrographis paniculata* [43], alleviates CUMS-induced depressive-like behaviors in rats by upregulating autophagy [44].

### 4.3 Saponins 

Saponins, naturally occurring compounds present in various plants and TCMs, exhibit a wide range of therapeutic effects [45]. Extracted from *Radix Paeoniae Rubra*, total paeony glycoside (TPG) restores mitochondrial function, inhibits inflammation-mediated pyroptosis, and activates autophagy to repair neuronal damage, all of which greatly aid antidepressant treatment [46]. The primary active ingredient in ginseng, Ginsenoside Rg1 (Rg1), has demonstrated potential in the prevention and treatment of neurological disorders, including depression [47]. According to Wang *et al*. [48], Rg1’s antidepressant action included modifying the *Cx43* autophagy-lysosomal and ubiquitin-proteasome pathways. Furthermore, by stimulating the BDNF-mTORC1 pathway, controlling autophagy, and improving hippocampus synaptic plasticity, ginseng total saponins and fuzi total alkaloids work in concert to produce antidepressant effects [49].

### 4.4 Polyphenols 

The human diet is abundant in polyphenols, a family of bioactive substances with phenolic structures [50]. Resveratrol is a key nutrient and a natural plant-derived antitoxin that helps plants defend against environmental stress and pathogen invasion [51]. Previous reports have shown that resveratrol can treat depression by regulating the hypothalamic-pituitary-adrenal (HPA) axis, alleviating neuroinflammation, and promoting neurogenesis [52]. In terms of regulating autophagy, Tabassum *et al*. [53] found that resveratrol (RSV) can regulate the expression of the Sirtuin-1 (SIRT1)/peroxisome proliferator-activated receptor gamma coactivator 1-alpha (PGC-1α)/Sirtuin-3 (SIRT3) signaling pathway, improve mitochondrial function and autophagy, and treat depression-like behavior caused by CUMS. Furthermore, by activating SIRT1, triggering autophagy, and blocking the AKT/mTOR signaling pathway, RSV can reduce postpartum depression-like behavior in mice [54].

### 4.5 Alkaloids

Alkaloids are naturally occurring metabolites with diverse regulatory effects on the body [55]. Alkaloids derived from TCM have been reported to have antidepressant effects. For instance, berberine, extracted from* Rhizoma coptidis*, has recently been found to exert antidepressant effects by inhibiting neuroinflammation, modulating neural plasticity, and regulating tryptophan metabolism [56, 57]. *Lotus plumule*, a traditional Chinese food, is rich in alkaloids, sterols, water-soluble polysaccharides, and various micronutrients, all of which provide numerous health benefits [58]. By controlling BDNF-mediated endoplasmic reticulum stress and autophagy, Chen *et al*. [59] showed via network pharmacology and experimental confirmation that natural alkaloids from lotus plumule mitigated LPS-induced depressive-like behavior.

### 4.6 Herbal Extracts

The only species in the genus Euryale within the botanical family *Nymphaeaceae* is the annual watery herbaceous plant *Euryale ferox* Salisb [60]. Extracts of *Euryale ferox* Salisb. can improve the AMPK pathway and correct autophagy abnormalities associated with depression, thereby alleviating depressive-like behavior induced by CUMS [61]. *Radix Polygalae*, a famous Chinese herbal medicine, has been widely used for centuries in traditional practices for its expectorant, nourishing, calming, and antipsychotic properties [62]. Evidence suggests that its active ingredient, polygalae radix oligosaccharide esters, can alleviate depression by regulating gut microbiota [63]. Additionally, in behavioral despair mice and CRS-induced rats, *Radix Polygalae* extract has shown strong antidepressant efficacy, most likely by enhancing autophagy and reducing neuroinflammation [64].

### 4.7 Other Types

**Fig. 1.**
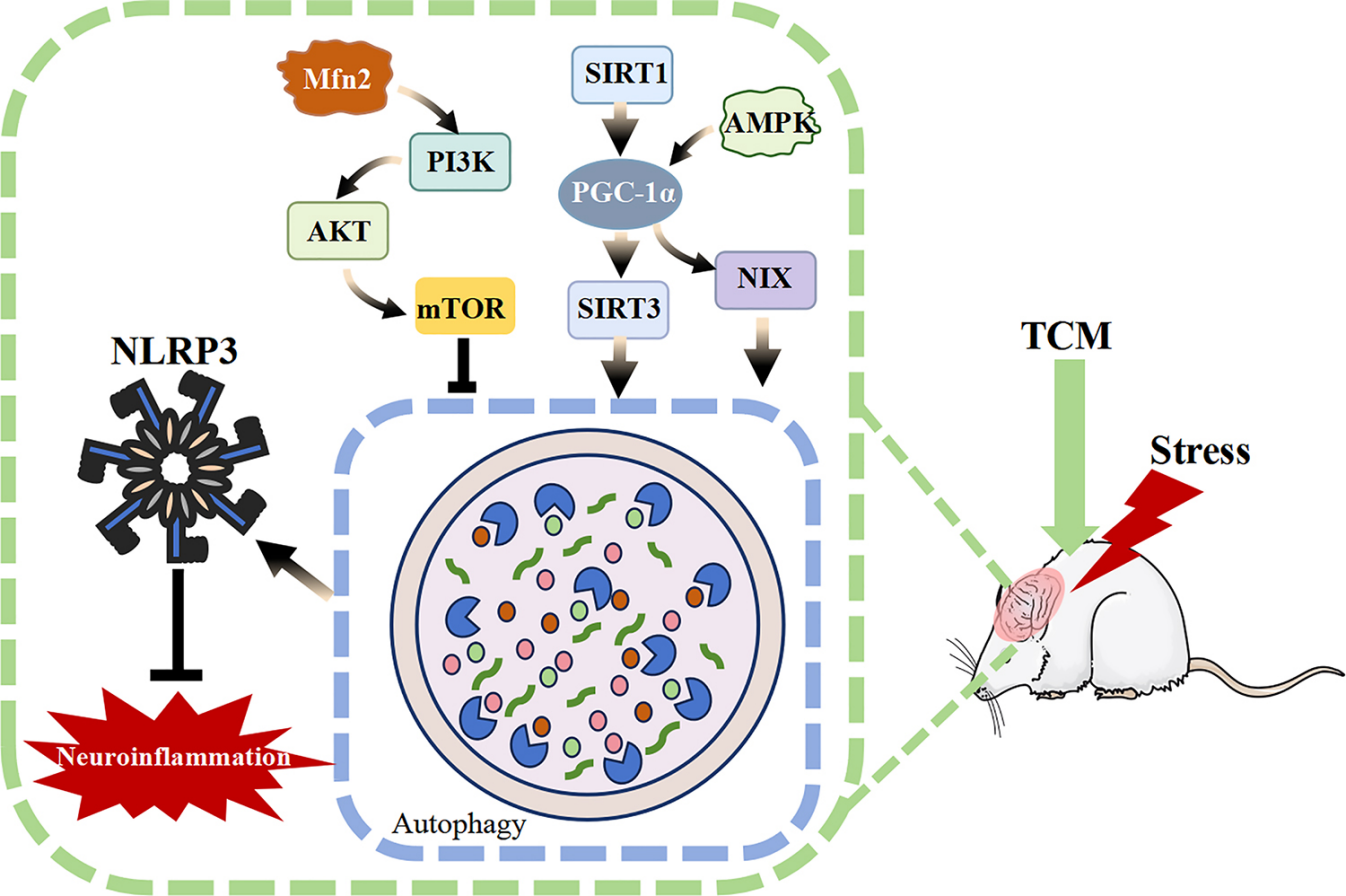
**Mechanism of action of active ingredients in traditional Chinese medicine**. AMPK, AMP-activated protein kinase; mTOR, mammalian target of rapamycin; SIRT1, sirtuin-1; SIRT3, sirtuin-3; NLRP3, NOD-like receptor pyrin domain-containing 3; TCM, traditional Chinese medicine; NIX, NIP3 like protein X; PI3K, phosphoinositide 3-kinase; AKT, protein kinase B; Mfn2, mitofusin 2 protein.

*Morinda officinalis *is one of the four famous Southern Chinese herbs, and its oligosaccharides are one of its main active ingredients. These oligosaccharides have been shown to treat depression by regulating the intestinal microbiota [65]. Recent studies have also found that *Morinda officinalis* oligosaccharides upregulate Mfn2 expression to activate the PI3K/Akt/mTOR pathway-mediated mitochondrial autophagy, thereby clearing damaged mitochondria in astrocytes and treating an animal model of hypertension with depression [66]. *Salvia miltiorrhiza *is one of the commonly used herbs in TCM and has attracted increasing interest in many medical areas [67]. It has been demonstrated that salvianolic acid B, an active component isolated from *Salvia miltiorrhiza*, controls autophagy and causes the clearance of NLRP3, resulting in neuroprotective and depressive effects (Fig. 1 and Table 1 (Ref. [28, 30, 33, 35, 36, 39, 42, 44, 46, 48, 53, 59, 61, 64, 66])).

**Table 1.** **Molecular mechanism of active ingredients in traditional Chinese medicine**.

Classification	Active ingredients of TCM	Molecular formula	CAS NO.	Main molecular mechanisms	References
Flavonoids	Quercetin	C_15_H_10_O_7_	117-39-5	Promoting mitochondrial autophagy to inhibit mtROS mediated NLRP3 inflammasome activation in microglia	[28]
	Baicalin	C_21_H_18_O_11_	21967-41-9	Activate AMPK/PGC-1α pathway enhancement of NIX mediated mitochondrial autophagy	[30]
	Apigenin	C_15_H_10_O_5_	520-36-5	Promoting autophagy through the AMPK/mTOR pathway	[33]
	Silibinin	C_25_H_22_O_10_	22888-70-6	Upregulation of BDNF/TrkB pathway activity has alleviated autophagy imbalance in the hippocampus	[35]
	Kaempferol-3-O-sophoroside	C_27_H_30_O_16_	19895-95-5	Combining with AMPK to promote BDNF production and enhance autophagy	[36]
Terpenoids	Oridonin	C_20_H_28_O_6_	28957-04-2	Blocking the interaction between NLRP3 and NEK7 to inhibit neuroinflammation and autophagy damage	[39]
	Patchouli alcohol	C_15_H_26_O	5986-55-0	Inhibiting autophagy, repairing synapses, and restoring autophagic flow in the hippocampus by activating the mTOR signaling pathway	[42]
	Andrographolide	C_20_H_30_O_5_	5508-58-7	Regulating autophagy	[44]
Saponins	Total paeony glycoside	Not Applicable	Not Applicable	Activating autophagy, restoring mitochondrial function, and reducing inflammation mediated pyroptosis to repair neuronal damage	[46]
	Ginsenoside Rg1	C_42_H_72_O_14_	22427-39-0	Regulating the ubiquitin proteasome and autophagy lysosomal degradation pathways of* Cx43*	[48]
Polyphenols	Resveratrol	C_14_H_12_O_3_	501-36-0	Regulating the expression of SIRT1/PGC-1α/SIRT3 signaling pathway, improving mitochondrial function and autophagy	[53]
Alkaloids	Natural alkaloids from *lotus plumule*	Not Applicable	Not Applicable	Regulating BDNF mediated endoplasmic reticulum stress and autophagy	[59]
Herbal extracts	Extract of *Euryale ferox* Salisb	Not Applicable	Not Applicable	Regulating the AMPK pathway to improve autophagy	[61]
	Radix Polygalae extract	Not Applicable	Not Applicable	Promoting autophagy and inhibiting neuroinflammation	[64]
Other types	*Morinda officinalis* oligosaccharides	Not Applicable	Not Applicable	Upregulation of Mfn2 expression to activate PI3K/Akt/mTOR pathway mediated mitochondrial autophagy	[66]

mtROS, mitochondrial ROS; BDNF, brain-derived neurotrophic factor; NEK7, NIMA related kinase 7; Cx43, connexin 43; PGC-1α, PPARγ coactivator 1-alpha.

## 5. Summary and Outlook

Major depressive disorder (MDD) is one of the most prevalent chronic, recurring, and incapacitating mental disorders. It is characterized by prolonged psychological distress, pain, feelings of worthlessness, sleep and appetite disturbances, and an increased risk of suicidal thoughts and behaviors [68]. Since its pathophysiology remains unclear and there are currently no sufficiently effective intervention strategies to successfully prevent or totally reverse the condition, MDD continues to provide a serious challenge to contemporary medicine [69]. TCM offers therapeutic benefits through its multi-component, multi-targets, and multi-pathway. Certain bioactive compounds extracted from TCM, such as flavonoids, terpenoids, saponins, polyphenols, alkaloids, herbal extracts, have been shown to exert antidepressant effects by regulating autophagy. These active ingredients hold significant potential for the treatment of MDD.

However, clinical studies investigating whether TCM’s active components can alleviate depression in MDD patients remain limited, with most research still confined to animal or cell models. Moreover, numerous TCM active substances face challenges such as low solubility, poor stability, and difficulty crossing the blood-brain barrier. Further research is needed to determine whether these active components can successfully target organs associated with MDD. The majority of research primarily focuses on molecular mechanisms, with insufficient scrutiny of the toxicological properties and potential adverse consequences of TCM’s active constituents. Furthermore, the stability and consistency of the chemical constituents in certain Chinese medicinal materials are compromised by ambiguous quality control requirements, thereby limiting their clinical applicability and complicating the investigation of their pharmacological actions. Moreover, the specificity of TCM’s active compounds in relation to autophagy remains inadequately elucidated, necessitating further research to ascertain whether these chemicals can precisely target autophagy. More critically, the conflicting findings regarding the increase or inhibition of neuronal autophagy in antidepressant therapy suggest that these results may be contingent upon the functional state of neurons during depression treatment.

## 6. Conclusion

Thus, clinical assessment of the effectiveness and side effects of TCM’s active components in treating MDD patients should be a primary focus of future research. Furthermore, comprehensive toxicity studies on TCM and the establishment of more rigorous quality control standards are imperative. To enhance the therapeutic effects of active components in target organs and further increase the concentration and duration of medications in the central nervous system, research on targeted delivery methods for TCM should also be intensified. That being said, our goal is to better understand the processes of depression and autophagy as well as the action routes of TCM by integrating spatial multi-omics approaches with single-cell sequencing. The mechanism by which TCM’s active components control autophagy should be elucidated using reverse validation techniques, such as the use of blockers or gene knockouts. Further preclinical and clinical research remains essential to comprehensively evaluate the impact of antidepressant treatments on autophagy, which is fundamental to the development of TCM-based antidepressants.

## Abbreviations

TCM, Traditional Chinese Medicine; SSRIs, selective serotonin reuptake inhibitors; HSC70, heat shock homologous protein 70; MDD, Major depressive disorder; mTOR, mammalian target of rapamycin; IL-1β, interleukin-1β; CRS, chronic restraint stress; NSCs, neural stem cells; CORT, corticosterone; ATG5, autophagy related gene 5; BDNF, brain-derived neurotrophic factor; AMPK, adenylate activated protein kinase; mtROS, mitochondrial reactive oxygen species; NEK7, NIMA related kinase 7; NLRP3, NOD like receptor pyridin domain containing 3; LPS, lipopolysaccharide; TPG, Total paeony glycoside; Rg1, Ginsenoside Rg1; PGC1α, peroxisome proliferator-activated receptor gamma coactivator 1-alpha; SIRT1, Sirtuin-1; SIRT3, Sirtuin-3.

## References

[1] Brown SJ, Huang XF, Newell KA (2021). The kynurenine pathway in major depression: What we know and where to next. *Neuroscience and Biobehavioral Reviews*.

[2] Bi C, Guo S, Hu S, Chen J, Ye M, Liu Z (2022). The microbiota-gut-brain axis and its modulation in the therapy of depression: Comparison of efficacy of conventional drugs and traditional Chinese medicine approaches. *Pharmacological Research*.

[3] Perez-Caballero L, Torres-Sanchez S, Bravo L, Mico JA, Berrocoso E (2014). Fluoxetine: a case history of its discovery and preclinical development. *Expert Opinion on Drug Discovery*.

[4] Rehm J, Shield KD (2019). Global Burden of Disease and the Impact of Mental and Addictive Disorders. *Current Psychiatry Reports*.

[5] Qu SY, Li XY, Heng X, Qi YY, Ge PY, Ni SJ (2021). Analysis of Antidepressant Activity of Huang-Lian Jie-Du Decoction Through Network Pharmacology and Metabolomics. *Frontiers in Pharmacology*.

[6] Wei Y, Chang L, Hashimoto K (2022). Molecular mechanisms underlying the antidepressant actions of arketamine: beyond the NMDA receptor. *Molecular Psychiatry*.

[7] Chi X, Wang S, Baloch Z, Zhang H, Li X, Zhang Z (2019). Research progress on classical traditional Chinese medicine formula Lily Bulb and Rehmannia Decoction in the treatment of depression. *Biomedicine & Pharmacotherapy = Biomedecine & Pharmacotherapie*.

[8] Fan L, Zeng P, Wang X, Mo X, Ma Q, Zhou X (2024). Xiaoyao Pills, a Chinese patent medicine, treats mild and moderate depression: A randomized clinical trial combined with DNA methylation analysis. *Phytomedicine: International Journal of Phytotherapy and Phytopharmacology*.

[9] Chen T, Tu S, Ding L, Jin M, Chen H, Zhou H (2023). The role of autophagy in viral infections. *Journal of Biomedical Science*.

[10] Levine B, Mizushima N, Virgin HW (2011). Autophagy in immunity and inflammation. *Nature*.

[11] Zhou B, Liu J, Kang R, Klionsky DJ, Kroemer G, Tang D (2020). Ferroptosis is a type of autophagy-dependent cell death. *Seminars in Cancer Biology*.

[12] Oku M, Sakai Y (2018). Three Distinct Types of Microautophagy Based on Membrane Dynamics and Molecular Machineries. *BioEssays: News and Reviews in Molecular, Cellular and Developmental Biology*.

[13] Dice JF (1990). Peptide sequences that target cytosolic proteins for lysosomal proteolysis. *Trends in Biochemical Sciences*.

[14] Ravanan P, Srikumar IF, Talwar P (2017). Autophagy: The spotlight for cellular stress responses. *Life Sciences*.

[15] Picca A, Faitg J, Auwerx J, Ferrucci L, D’Amico D (2023). Mitophagy in human health, ageing and disease. *Nature Metabolism*.

[16] Alcocer-Gómez E, Casas-Barquero N, Núñez-Vasco J, Navarro-Pando JM, Bullón P (2017). Psychological status in depressive patients correlates with metabolic gene expression. *CNS Neuroscience & Therapeutics*.

[17] Machado-Vieira R, Zanetti MV, Teixeira AL, Uno M, Valiengo LL, Soeiro-de-Souza MG (2015). Decreased AKT1/mTOR pathway mRNA expression in short-term bipolar disorder. *European Neuropsychopharmacology: the Journal of the European College of Neuropsychopharmacology*.

[18] Jernigan CS, Goswami DB, Austin MC, Iyo AH, Chandran A, Stockmeier CA (2011). The mTOR signaling pathway in the prefrontal cortex is compromised in major depressive disorder. *Progress in Neuro-psychopharmacology & Biological Psychiatry*.

[19] Osborne LM, Payne JL, Sherer ML, Sabunciyan S (2022). Altered extracellular mRNA communication in postpartum depression is associated with decreased autophagy. *Molecular Psychiatry*.

[20] He S, Deng Z, Li Z, Gao W, Zeng D, Shi Y (2021). Signatures of 4 autophagy-related genes as diagnostic markers of MDD and their correlation with immune infiltration. *Journal of Affective Disorders*.

[21] Li MM, Wang X, Chen XD, Yang HL, Xu HS, Zhou P (2022). Lysosomal dysfunction is associated with NLRP3 inflammasome activation in chronic unpredictable mild stress-induced depressive mice. *Behavioural Brain Research*.

[22] Fu J, Wu H (2023). Structural Mechanisms of NLRP3 Inflammasome Assembly and Activation. *Annual Review of Immunology*.

[23] Jung S, Choe S, Woo H, Jeong H, An HK, Moon H (2020). Autophagic death of neural stem cells mediates chronic stress-induced decline of adult hippocampal neurogenesis and cognitive deficits. *Autophagy*.

[24] Zhang K, Wang F, Zhai M, He M, Hu Y, Feng L (2023). Hyperactive neuronal autophagy depletes BDNF and impairs adult hippocampal neurogenesis in a corticosterone-induced mouse model of depression. *Theranostics*.

[25] Li Y, Cheng Y, Zhou Y, Du H, Zhang C, Zhao Z (2022). High fat diet-induced obesity leads to depressive and anxiety-like behaviors in mice via AMPK/mTOR-mediated autophagy. *Experimental Neurology*.

[26] Ku YS, Ng MS, Cheng SS, Lo AWY, Xiao Z, Shin TS (2020). Understanding the Composition, Biosynthesis, Accumulation and Transport of Flavonoids in Crops for the Promotion of Crops as Healthy Sources of Flavonoids for Human Consumption. *Nutrients*.

[27] Hosseini A, Razavi BM, Banach M, Hosseinzadeh H (2021). Quercetin and metabolic syndrome: A review. *Phytotherapy Research: PTR*.

[28] Han X, Xu T, Fang Q, Zhang H, Yue L, Hu G (2021). Quercetin hinders microglial activation to alleviate neurotoxicity via the interplay between NLRP3 inflammasome and mitophagy. *Redox Biology*.

[29] Wen RJ, Dong X, Zhuang HW, Pang FX, Ding SC, Li N (2023). Baicalin induces ferroptosis in osteosarcomas through a novel Nrf2/xCT/GPX4 regulatory axis. *Phytomedicine: International Journal of Phytotherapy and Phytopharmacology*.

[30] Jin X, Zhu L, Lu S, Li C, Bai M, Xu E (2023). Baicalin ameliorates CUMS-induced depression-like behaviors through activating AMPK/PGC-1α pathway and enhancing NIX-mediated mitophagy in mice. *European Journal of Pharmacology*.

[31] Oh HM, Cho CK, Lee NH, Son CG (2024). Experimental evidence for anti-metastatic actions of apigenin: a mini review. *Frontiers in Oncology*.

[32] Zhang L, Lu RR, Xu RH, Wang HH, Feng WS, Zheng XK (2023). Naringenin and apigenin ameliorates corticosterone-induced depressive behaviors. *Heliyon*.

[33] Zhang X, Bu H, Jiang Y, Sun G, Jiang R, Huang X (2019). The antidepressant effects of apigenin are associated with the promotion of autophagy via the mTOR/AMPK/ULK1 pathway. *Molecular Medicine Reports*.

[34] Bosch-Barrera J, Queralt B, Menendez JA (2017). Targeting STAT3 with silibinin to improve cancer therapeutics. *Cancer Treatment Reviews*.

[35] Song X, Liu B, Cui L, Zhou B, Liu W, Xu F (2017). Silibinin ameliorates anxiety/depression-like behaviors in amyloid β-treated rats by upregulating BDNF/TrkB pathway and attenuating autophagy in hippocampus. *Physiology & Behavior*.

[36] Wang R, Hu X, Liu S, Wang J, Xiong F, Zhang X (2024). Kaempferol-3-O-sophoroside (PCS-1) contributes to modulation of depressive-like behaviour in C57BL/6J mice by activating AMPK. *British Journal of Pharmacology*.

[37] Bergman ME, Davis B, Phillips MA (2019). Medically Useful Plant Terpenoids: Biosynthesis, Occurrence, and Mechanism of Action. *Molecules (Basel, Switzerland)*.

[38] Li X, Zhang CT, Ma W, Xie X, Huang Q (2021). Oridonin: A Review of Its Pharmacology, Pharmacokinetics and Toxicity. *Frontiers in Pharmacology*.

[39] Liang L, Wang H, Hu Y, Bian H, Xiao L, Wang G (2022). Oridonin relieves depressive-like behaviors by inhibiting neuroinflammation and autophagy impairment in rats subjected to chronic unpredictable mild stress. *Phytotherapy Research: PTR*.

[40] Li C, Zhu Y, Wu Y, Fu M, Wu Y, Wu Y (2022). Oridonin Alleviates LPS-Induced Depression by Inhibiting NLRP3 Inflammasome via Activation of Autophagy. *Frontiers in Medicine*.

[41] He H, Xie X, Zhang J, Mo L, Kang X, Zhang Y (2023). Patchouli alcohol ameliorates depression-like behaviors through inhibiting NLRP3-mediated neuroinflammation in male stress-exposed mice. *Journal of Affective Disorders*.

[42] Zhuo J, Chen B, Sun C, Jiang T, Chen Z, Liu Y (2020). Patchouli alcohol protects against chronic unpredictable mild stress-induced depressant-like behavior through inhibiting excessive autophagy via activation of mTOR signaling pathway. *Biomedicine & Pharmacotherapy = Biomedecine & Pharmacotherapie*.

[43] Zeng B, Wei A, Zhou Q, Yuan M, Lei K, Liu Y (2022). Andrographolide: A review of its pharmacology, pharmacokinetics, toxicity and clinical trials and pharmaceutical researches. *Phytotherapy Research: PTR*.

[44] Geng J, Liu J, Yuan X, Liu W, Guo W (2019). Andrographolide triggers autophagy-mediated inflammation inhibition and attenuates chronic unpredictable mild stress (CUMS)-induced depressive-like behavior in mice. *Toxicology and Applied Pharmacology*.

[45] Zhang R, Zeng M, Zhang X, Zheng Y, Lv N, Wang L (2023). Therapeutic Candidates for Alzheimer’s Disease: Saponins. *International Journal of Molecular Sciences*.

[46] Su L, Lu H, Zhang D, Zhu X, Li J, Zong Y (2024). Total paeony glycoside relieves neuroinflammation to exert antidepressant effect via the interplay between NLRP3 inflammasome, pyroptosis and autophagy. *Phytomedicine: International Journal of Phytotherapy and Phytopharmacology*.

[47] Yang SJ, Wang JJ, Cheng P, Chen LX, Hu JM, Zhu GQ (2023). Ginsenoside Rg1 in neurological diseases: From bench to bedside. *Acta Pharmacologica Sinica*.

[48] Wang HQ, Yang SW, Gao Y, Liu YJ, Li X, Ai QD (2021). Novel antidepressant mechanism of ginsenoside Rg1: Regulating biosynthesis and degradation of connexin43. *Journal of Ethnopharmacology*.

[49] Jin Y, Pang H, Zhao L, Zhao F, Cheng Z, Liu Q (2022). Ginseng total saponins and Fuzi total alkaloids exert antidepressant-like effects in ovariectomized mice through BDNF-mTORC1, autophagy and peripheral metabolic pathways. *Phytomedicine: International Journal of Phytotherapy and Phytopharmacology*.

[50] Guo Y, Li Z, Chen F, Chai Y (2023). Polyphenols in Oral Health: Homeostasis Maintenance, Disease Prevention, and Therapeutic Applications. *Nutrients*.

[51] Ren B, Kwah MXY, Liu C, Ma Z, Shanmugam MK, Ding L (2021). Resveratrol for cancer therapy: Challenges and future perspectives. *Cancer Letters*.

[52] Moore A, Beidler J, Hong MY (2018). Resveratrol and Depression in Animal Models: A Systematic Review of the Biological Mechanisms. *Molecules (Basel, Switzerland)*.

[53] Tabassum S, Misrani A, Huang HX, Zhang ZY, Li QW, Long C (2023). Resveratrol Attenuates Chronic Unpredictable Mild Stress-Induced Alterations in the SIRT1/PGC1α/SIRT3 Pathway and Associated Mitochondrial Dysfunction in Mice. *Molecular Neurobiology*.

[54] Ye S, Fang L, Xie S, Hu Y, Chen S, Amin N (2023). Resveratrol alleviates postpartum depression-like behavior by activating autophagy via SIRT1 and inhibiting AKT/mTOR pathway. *Behavioural Brain Research*.

[55] Bhambhani S, Kondhare KR, Giri AP (2021). Diversity in Chemical Structures and Biological Properties of Plant Alkaloids. *Molecules (Basel, Switzerland)*.

[56] Qin Z, Shi DD, Li W, Cheng D, Zhang YD, Zhang S (2023). Berberine ameliorates depression-like behaviors in mice via inhibiting NLRP3 inflammasome-mediated neuroinflammation and preventing neuroplasticity disruption. *Journal of Neuroinflammation*.

[57] Ge PY, Qu SY, Ni SJ, Yao ZY, Qi YY, Zhao X (2023). Berberine ameliorates depression-like behavior in CUMS mice by activating TPH1 and inhibiting IDO1-associated with tryptophan metabolism. *Phytotherapy Research: PTR*.

[58] Liu B, Li J, Yi R, Mu J, Zhou X, Zhao X (2019). Preventive Effect of Alkaloids from Lotus plumule on Acute Liver Injury in Mice. *Foods (Basel, Switzerland)*.

[59] Chen S, Guo W, Qi X, Zhou J, Liu Z, Cheng Y (2019). Natural alkaloids from lotus plumule ameliorate lipopolysaccharide-induced depression-like behavior: integrating network pharmacology and molecular mechanism evaluation. *Food & Function*.

[60] Liu X, He Z, Yin Y, Xu X, Wu W, Li L (2018). Transcriptome sequencing and analysis during seed growth and development in Euryale ferox Salisb. *BMC Genomics*.

[61] Huang Z, Huang X, Wang Q, Jiang R, Sun G, Xu Y (2018). Extract of Euryale ferox Salisb exerts antidepressant effects and regulates autophagy through the adenosine monophosphate-activated protein kinase-UNC-51-like kinase 1 pathway. *IUBMB Life*.

[62] Jiang N, Wei S, Zhang Y, He W, Pei H, Huang H (2021). Protective Effects and Mechanism of Radix Polygalae Against Neurological Diseases as Well as Effective Substance. *Frontiers in Psychiatry*.

[63] Chen Q, Jia T, Wu X, Chen X, Wang J, Ba Y (2023). Polygalae Radix Oligosaccharide Esters May Relieve Depressive-like Behavior in Rats with Chronic Unpredictable Mild Stress via Modulation of Gut Microbiota. *International Journal of Molecular Sciences*.

[64] Zhou Y, Yan M, Pan R, Wang Z, Tao X, Li C (2021). Radix Polygalae extract exerts antidepressant effects in behavioral despair mice and chronic restraint stress-induced rats probably by promoting autophagy and inhibiting neuroinflammation. *Journal of Ethnopharmacology*.

[65] Zhang ZW, Gao CS, Zhang H, Yang J, Wang YP, Pan LB (2022). Morinda officinalis oligosaccharides increase serotonin in the brain and ameliorate depression via promoting 5-hydroxytryptophan production in the gut microbiota. *Acta Pharmaceutica Sinica. B*.

[66] Yang L, Ao Y, Li Y, Dai B, Li J, Duan W (2023). Morinda officinalis oligosaccharides mitigate depression-like behaviors in hypertension rats by regulating Mfn2-mediated mitophagy. *Journal of Neuroinflammation*.

[67] Jia Q, Zhu R, Tian Y, Chen B, Li R, Li L (2019). Salvia miltiorrhiza in diabetes: A review of its pharmacology, phytochemistry, and safety. *Phytomedicine: International Journal of Phytotherapy and Phytopharmacology*.

[68] Xia CY, Guo YX, Lian WW, Yan Y, Ma BZ, Cheng YC (2023). The NLRP3 inflammasome in depression: Potential mechanisms and therapies. *Pharmacological Research*.

[69] Chen J, Lei C, Li X, Wu Q, Liu C, Ma Q (2022). Research progress on classical traditional chinese medicine formula xiaoyaosan in the treatment of depression. *Frontiers in Pharmacology*.

